# Reappraising suppression: subjective and physiological correlates of experiential suppression in healthy adults

**DOI:** 10.3389/fpsyg.2014.00571

**Published:** 2014-06-11

**Authors:** Mathieu Lemaire, Wissam El-Hage, Sophia Frangou

**Affiliations:** ^1^Université François-Rabelais de Tours–INSERM “Imagerie et Cerveau” UMR U 930Tours, France; ^2^Centre Universitaire de Pédopsychiatrie, Centre Hospitalier Régional Universitaire de ToursTours, France; ^3^Centre d’Investigation Clinique, INSERM 1415Tours, France; ^4^Clinique Psychiatrique Universitaire, Centre Hospitalier Régional Universitaire de ToursTours, France; ^5^Psychosis Research Program, Department of Psychiatry, Icahn School of Medicine at Mount SinaiNew York, NY, USA

**Keywords:** emotion regulation, experiential suppression, skin conductance response, heart rate, time course

## Abstract

**Background:** Emotion regulation strategies based on suppressing behavioral expressions of emotion have been considered maladaptive. However, this may not apply to suppressing the emotional experience (experiential suppression). The aim of this study was to define the effect of experiential suppression on subjective and physiological emotional responses.

**Methods:** Healthy adults (*N* = 101) were characterized in terms of the temperament, personality, and hedonic capacity using the Tridimensional Personality Questionnaire, the Eysenck Personality Questionnaire, the Fawcett–Clark Pleasure Scale, and the State-Trait Anxiety Inventory. Participants were shown positive, negative, and neutral pictures from the International Affective Picture System under two conditions, passive viewing, and experiential suppression. During both conditions, subjective ratings of the intensity and duration of emotional responses and physiological measures of skin conductance (SC) and cardiac inter-beat interval (IBI) to each picture were recorded.

**Results:** Negative pictures elicited the most intense physiological and emotional responses regardless of experimental condition. Ratings of emotional intensity were not affected by condition. In contrast, experiential suppression, compared to passive viewing, was associated with decreased duration of the emotional response, reduced maximum SC amplitude and longer IBIs independent of age, picture valence, personality traits, hedonic capacity, and anxiety.

**Conclusion:** These findings demonstrate that experiential suppression may represent an adaptive emotion regulation mechanism associated with reduced arousal and cardiovascular activation.

## INTRODUCTION

Emotion regulation refers to processes by which we monitor, evaluate, and modify emotions according to the context in which they occur (Gross and Barrett, 2011). The ability to successfully regulate emotions is associated with positive psychosocial and health outcomes (Damasio and Carvalho, 2013). Conversely deficits in emotion regulation are integral to the development and maintenance of a wide range of mental disorders (e.g., Karevold et al., 2009; Mennin et al., 2009; Perry et al., 2012).

Prevalent models of emotion regulation distinguish between strategies that modify emotional triggers (antecedent-focused strategies) and those that modulate the response to an emotion once it has arisen (response-focused strategies; Gross, 1998). Suppression is considered a key response-focused strategy that can be deployed to inhibit either emotional experience (experiential suppression; Quartana and Burns, 2007), emotion-related thoughts (thought suppression; Wenzlaff and Wegner, 2000), or overt emotion-related behavior (expressive suppression; Gross, 1998). Expressive suppression has been studied extensively and has been consistently associated with increased levels of psychopathology (Aldao et al., 2010; Wolgast et al., 2013), heightened sympathetic response (Webb et al., 2012; Dan-Glauser and Gross, 2013) particularly in western cultures (Zhou and Bishop, 2012), minimal (Webb et al., 2012; Dan-Glauser and Gross, 2013), or decreased impact on emotion experience (Gross and Levenson, 1993, 1997; Gross, 2002; Butler et al., 2013) and adverse cognitive effects (Hayes et al., 2010; Moore and Zoellner, 2012). Thought suppression has been associated with paradoxical increase in the accessibility of the suppressed thoughts, enhancement of the associated emotion and sympathetic arousal (Wenzlaff and Wegner, 2000).

Based on this evidence suppression is considered a maladaptive emotion regulation strategy. An implicit but largely untested assumption is that this also applies to experiential suppression. In a recent review of 190 studies on emotion regulation, Webb et al. (2012) identified only four studies on experiential suppression. In these studies, experiential suppression did not have any adverse influence on measures of cardiovascular activation, anxiety, or general distress (Burns et al., 2007; Quartana and Burns, 2010). However, there has since been renewed interest in contrasting experiential and expressive suppression. Dan-Glauser and Gross (2011) contrasted expressive to “physiological” suppression, the later requiring participants to suppress bodily reactions to emotional stimuli by focusing on their breathing. It is not clear whether participants perceived these are two strategies as different as their expressivity was equally reduced to minimal in both conditions. The only difference was that physiological suppression was more effective in down-regulating emotional and cardiovascular responses to positive stimuli. Moore and Zoellner (2012) compared experiential to expressive suppression in women and found an advantage for experiential suppression in reducing the experience of negative emotion and associated distress. Similarly, health individuals in another study were able to down regulate their cardiovascular response of experimentally delivered carbon dioxide (Kashdan et al., 2006). It is therefore possible that experiential suppression may represent as more advantageous emotion regulation strategy compared to expressive suppression.

The aim of the present study was to define the effect of experiential suppression on subjective ratings of emotional intensity and duration and on objective measures of arousal and cardiovascular activation. Healthy adults (*N* = 101) were presented with positive, negative, and neutral pictures from the International Affective Picture System (Lang et al., 2008) under two conditions, passive viewing, and experiential suppression. Skin conductance (SC) was used to index sympathetic activity and cardiac inter-beat interval (IBI) was used to index parasympathetic activity (Bradley et al., 2008; Kreibig, 2010; Billman, 2011). Our tentative hypotheses were that experiential suppression would be associated with down regulation of emotional experience without concomitant increase in measures of arousal. We further tested whether subjective and objective measures of experiential suppression were modulated by stimulus valence and personality characteristics.

## MATERIALS AND METHODS

### PARTICIPANTS

Healthy Caucasian, native English speakers, aged 25–65 years, were recruited from the local community through advertisements and assessed with the non-patient version of the Structural Clinical Interview for Diagnostic and Statistical Manual of Mental Disorders (DSM-IV) Axis I disorders (First et al., 2007). They were excluded if they had (i) lifetime history of Axis I disorder or substance abuse as defined by the DSM-IV-TR [American Psychiatric Association (APA), 2000], (ii) regular prescribed medication excluding contraceptives, (iii) concurrent medical or neurological conditions or history of head injury, (iv) family history of hereditary central nervous system disorders, (v) verbal intelligence quotient (IQ) < 75 based on the vocabulary subscale of Wechsler Adult Intelligence Scale-Revised (WAIS-R; Wechsler, 1997). We estimated using G^*^Power 3.1.5^1^) that a sample of 101 individuals would be sufficient to detect univariate correlations as low as 0.3 with a conservative alpha = 0.01 and 80% power. The study was approved by the local review board and written informed consent was obtained from all participants according to the Declaration of Helsinki.

### ASSESSMENT OF PERSONALITY, HEDONIC CAPACITY, AND STATE-TRAIT ANXIETY

Participants completed the following questionnaires prior to the experiment:

(a) Tridimensional Personality Questionnaire (TPQ; Cloninger et al., 1991), a 100-item self-report questionnaire that generates three temperament scales: novelty seeking (TPQ-NS), harm avoidance (TPQ-HA), and reward dependence (TPQ-RD).
(b) Eysenck Personality Questionnaire (EPQ; Eysenck and Eysenck, 1975), a 90-item self-report questionnaire that measures Psychoticism (EPQ-P), Extraversion (EPQ-E), and Neuroticism (EPQ-N). It also includes a validity index, the Lie scale, which is an indicator of social desirability.
(c) Fawcett–Clark Pleasure Scale (FCPS; Fawcett et al., 1983), a 36 self-report item scale that covers different domains of hedonic experience, including social activities, sensory experiences, and sense of mastery of difficult tasks. Higher FCPS total scores indicate greater hedonic capacity.
(d) State-Trait Anxiety Inventory (STAI; Spielberger, 1983) which rates state (STAI-S) and trait anxiety (STAI-T).

### EXPERIMENTAL DESIGN

Following screening for eligibility, participants were tested individually in a single experimental session that comprised a familiarization session, and two experimental conditions (passive viewing and experiential suppression). They were seated in a comfortable semi-reclining chair in a dimly lit and sound-attenuated room next to an observation room. They were told that during the experiment, pictures of emotional content would be presented on the screen and that each picture should be viewed for the entire presentation time. Prior to testing, six pictures (four neutral, one positive, and one negative), not shown later in the experiment, were used to familiarize participants with the procedure. Data from these pictures were not included in the analyses.

#### Emotional stimuli

Positive, negative, and neutral pictures (*N* = 108) were selected from the International Affective Picture System (IAPS) on the basis of normative subjective ratings of valence and arousal (Lang et al., 2008). Valence ratings above 7 or below 3 were, respectively, used to identify positive and negative pictures while an average valence rating of 5 (0.5) was used for neutral pictures. Regardless of positive or negative valence, pictures were also selected on the basis of high (≥6) normative arousal ratings (**Table 1**). Positive images depicted adventure scenes (e.g., cliff diving, mountain climbing), negative images portrayed scenes of violence (e.g., aggression, physical brutality, and combat), or threatening figures or weapons (e.g., pointed guns, looming attackers). Neutral pictures showed buildings and household objects. Normative arousal ratings did not differ between negative and positive pictures (*p* > 0.05). The relevance of the normative valence ratings to this study was confirmed by asking participants to rate all stimuli they had viewed at the end of the experimental procedures described below. The analysis of valence ratings yielded results consistent with the normative IAPS (Lang et al., 2008) ratings within each category. Mean (SD) valence ratings given were as follows: 2.16 (0.56) for negative pictures, 7.01 (0.64) for positive pictures and 5.10 (0.25) for neutral pictures on the SAM scale. As expected, negative pictures were rated less pleasant and positive images more pleasant than neutral ones (all *p* < 0.0001); negative images were statistically less pleasant than positive ones (*p* < 0.0001).

**Table 1:** Normative data for valence and arousal for IAPS pictures.

Pictures	Valence	Arousal
**Set 1**
Negative	2.19 (1.47)	6.11 (1.95)
Positive	7.39 (1.38)	6.48 (1.91)
Neutral	4.87 (1.01)	2.32 (1.73)
**Set 2**
Negative	2.34 (1.55)	6.29 (2.24)
Positive	7.39 (1.37)	6.10 (1.91)
Neutral	4.87 (1.01)	2.32 (1.73)

*Data are shown as mean (SD); IAPS Picture numbers were as follows: Set 1: negative pictures: 3010, 3170, 3180, 3500, 6212, 6230, 6313, 6360, 6370, 6510, 6821, 6830, 9006, 9050, 9253, 9410, 9611, 9921; positive pictures: 8300, 4640, 5270, 5470, 8034, 8080, 8090, 8170, 8180, 8190, 8370, 8380, 8400, 8470, 8496, 8501, 8531, 4607. Set 2: negative pictures: 3063, 3550, 6250, 6300, 6350, 6560, 6570, 6831, 8230, 9250, 9252, 9400, 9420, 9600, 9630, 9800, 9810, 9910; positive pictures: 4599, 4608, 4660, 5260,5450, 5460, 5480, 5621, 5623, 5629, 5700, 5910, 7502, 8030, 8200, 8210, 8420, 8490; neutral pictures (common between two sets): 7090, 7004, 7006, 7010, 7020, 7031, 7035, 7040, 7060, 7080, 7110, 7150, 7175, 7185, 7187, 7217, 7491, 7000.
*

#### Experimental conditions

**FIGURE 1:**
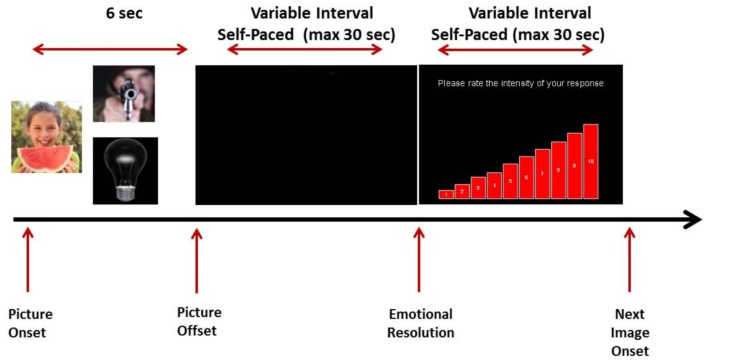
**Experimental procedure**.

***Passive viewing***. Participants were shown 54 IAPS pictures equally split into positive, negative, and neutral. The order of image presentation was randomized across individuals. Pictures were presented through an Epson image projector onto a blank wall at a distance of approximately 2 m from the participants. Each picture was presented for 6 s and was followed by a blank screen. Participants were instructed to simply view the images and indicate by button press when their emotional response to the picture they had just seen had completely subsided (time to emotional resolution). Immediately afterwards participants were presented with a new screen depicting an ascending bar chart ranging from 1 to 10; 1 indicated “minimal emotional response” while 10 indicated an “extremely intense” emotional response. They were asked to rate by clicking with the mouse on the corresponding point of the scale the maximal intensity of their emotional reaction to the image they had just seen. Participants’ assessment was obtained after response resolution as directing attention to one’s emotional state is known to augment intensity (Lane et al., 1997). After rating the intensity of their emotional response, participants were then presented with the next IAPS picture. A schematic representation of the paradigm is shown in **Figure 1**.

At the beginning of the passive condition the following instructions were presented on screen:

“You are going to see a number of images (one at a time) that may make you feel various different emotions. I want you to look at them and allow yourself to naturally experience any emotional reaction to these images. After each image you will see a black screen. During this black screen, I want you to press the spacebar when you feel that the emotion from the image you previously saw is gone. In other words, you need to wait until your response fades away and then press the spacebar. After pressing the spacebar, you will be asked to rate the intensity of the emotion you felt while viewing the image by clicking with the mouse on a scale that ranges from 1 (minimal intensity) to 10 (extremely high intensity): for example, clicking on the lowest bar would mean that the image had a minimal emotional effect on you while clicking on the highest bar would suggest that the images had a major emotional effect on you. Please remember that this task does not require any effort from you in order change your emotional response in any way: you may take as much time as you need before pressing the spacebar.”

***Experiential suppression***. The experimental set-up for the suppression condition was identical to that of the passive viewing condition with two exceptions; participants were shown a different set of 54 IAPS pictures and were instructed to suppress their emotional reaction to each picture presented and indicate by button press when that was accomplished.

At the beginning of the suppression condition participants were given the following instructions:

“You are going to see a number of images (one at a time) that may make you feel various different emotions. I want you to look at them very carefully allow yourself to feel whatever you see in these images each time. After each image you will see a black screen. During this black screen period, I want you to regulate any emotion you felt from viewing the image. Please press the spacebar when you feel that the emotion from the image you previously saw has been successfully suppressed by you. After this, you will be asked to rate the intensity of the emotion you felt while viewing the image by clicking with the mouse on a scale that ranges from 1 (minimal intensity) to 10 (extremely high intensity): for example, clicking on the lowest bar would mean that the image had a minimal emotional effect on you while clicking on the highest bar would suggest that it had a major emotional effect on you. Please remember that this is an active task that requires effort from you in order to make the emotional response go away; you may take as much time as you need before pressing the spacebar.”

The picture sets used were counterbalanced across conditions and conditions were counterbalanced between participants. For both conditions, self-report measures analyzed were participants’ ratings of the intensity of their emotional response to each picture and time to emotional resolution for each picture. Time to emotional resolution was defined as the period between stimulus offset and participants indicating the end of their emotional response.

### PHYSIOLOGICAL MEASURES

Skin conductance and electrocardiogram (ECG) were recorded using SONY CONTACT PSYLAB Stand Alone Monitor unit^2^ programmed to be compatible with Visual Basic Software (Visual Basic 6.0).

#### Skin conductance

Skin conductance was constantly acquired by a SC5 digital amplifier (24 bit resolution) using a constant voltage (0.5 V) method from the index and middle fingers of the non-dominant hand. A pair of pre-wired Ag/AgCl electrodes were filled with 0.5% Mansfield electrode paste and collars (TD-22 EL1, Med Associates Inc., St Albans, VT, USA) were used to give a constant area of 0.8 mm per electrode (Fowles et al., 1981). The channels were defined as follows: Sampling rate 1000 Hz, Hz idling 100, graph speed % of max 100, range 1 mV, high pass filter 10 Hz, low pass filter 40 Hz, buffer size 1024. The accuracy of SC measurement was checked with the two calibrator buttons [20 and 0.1 μS] provided in the pre-amplifier.

The experiment started with a 15-min idling period in order to calibrate participants’ responses before SC recordings began. The signal was sampled at a rate of 100 Hz per second, measured into a SC value (in μS) and saved for later analysis. Raw data were then converted into standard ASCII format files with PSYLAB-8 software^3^ and then into numeric values for further analysis. All recordings were screened visually for possible artifacts.

Skin conductance responses (SCRs) were defined as local maxima with minimum amplitude of 0.01 μS and minimum rise time of 500 ms (Fowles et al., 1981; Boucsein et al., 2012). For each image the amplitude of the maximum SCR peak (maximum SC amplitude) was measured from image onset until participants indicated the end of the associated emotional response by button press. Maximum SC amplitude refers to the maximum level reached compared to the start of the SC response. In each condition (passive and suppression) the maximum SC amplitude were averaged separately within each valence.

#### Inter-beat interval

Inter-beat interval refers to the interval, in milliseconds, between successive heart contractions (R waves of the ECG). We recorded participants’ ECG using three disposable electrodes each attached to the chest (beneath the right and left clavicle) and upper left abdominal quadrant. Raw data were converted into standard ASCII format files with PSYLAB-8 software. A Schmitt trigger was used to mark the occurrence of each R wave, and record IBI to the nearest millisecond. IBIs were averaged for each valence within each condition (passive and suppression).

### DATA ANALYSIS

The normality of distribution of all variables was examined and logarithmic transformation was required for time to emotional resolution and maximum SC amplitude. First, we examined the possible effect of task order. We compared subjective and physiological measures in the passive viewing condition between individuals that underwent the suppression condition before or after the passive viewing condition. Second, we explored the univariate correlations between subjective and physiological measures separately for each condition. We then used mixed-effects regression models to assess the effect of valence (negative, neutral, and positive), condition (passive, suppression), sex, age, personality, and anxiety measures on psychophysiological data (maximum SCR amplitude and IBI), subjective ratings of intensity and time to emotional resolution. The best fitting model was selected following step-by-step elimination of non-significant measures from previous models. Valence, condition, sex, age were forced in the model even if they were not significant. Fitted models were inspected graphically to confirm the underlying distributional assumptions (Pinheiro and Bates, 2009). All statistics analyses were implemented in R 2.15.1^4^. The mixed models were fitted using “nlme” R package (Pinheiro et al., 2012). For factors showing a significant main fixed effect, pairwise comparisons were conducted with Bonferroni correction using “multcomp” R package (Hothorn et al., 2008).

## RESULTS

One hundred and one participants (49 females, 52 males) aged 25–63 years were enrolled (**Table 2**). Their mean age was 43.9 (11.9) years and their mean IQ was 102.1 (15.5). The means and SDs of time to emotional resolution, subjective ratings of emotional intensity, maximum SC amplitude, and IBI for positive, negative, and neutral pictures in each condition are reported in **Table 3**. No order of condition effect was observed (all pairwise comparisons *p* > 0.34).

**Table 2:** *S*ample characteristics (*N* = 101).

	Mean (SD)	Range
STAI-S	28.7 (6.9)	20–49
STAI-T	34.7 (8.9)	20–55
TPQ-NS	17.0 (5.7)	5–30
TPQ-HA	11.4 (6.4)	2–28
TPQ-RD	17.0 (5.1)	5–28
FCPS	136.2 (15.0)	102–171
EPQ-E	13.6 (5.0)	2–21
EPQ-N	8.8 (5.2)	1–20
EPQ-P	3.6 (2.8)	0–14

*SD: Standard Deviation; STAI: State-Trait Anxiety Inventory; STAI-S: state anxiety; STAI-T: trait anxiety; TPQ: Tridimensional Personality Questionnaire; TPQ-NS: novelty seeking; TPQ-HA: harm avoidance; TPQ-RD: reward dependence; FCPS: Fawcett–Clark Pleasure Scale; EPQ: Eysenck Personality Questionnaire; EPQ-E: Extraversion; EPQ-N: Neuroticism; EPQ-P: Psychoticism.*

**Table 3:** Subjective ratings and physiological measures per condition and picture valence in the study sample (*N* = 101).

Valence	Time to emotional resolution	Emotional intensity ratings	Maximum SC amplitude	Inter-beat interval
**Passive condition**
Positive	11.01 (6.57)	5.65 (1.80)	0.19 (0.14)	854.99 (115.50)
Negative	14.74 (7.82)	7.45 (1.71)	0.21 (0.17)	857.53 (114.14)
Neutral	5.27 (3.75)	1.77 (0.87)	0.16 (0.12)	855.38 (123.36)
**Experiential suppression condition**
Positive	8.05 (5.81)	5.74 (1.87)	0.16 (0.13)	863.93 (113.46)
Negative	10.52 (6.40)	7.23 (1.76)	0.18 (0.17)	865.92 (117.88)
Neutral	4.54 (4.23)	1.71 (0.82)	0.15 (0.14)	858.73 (115.14)

*Data are presented as mean (SD); SC = skin conductance; time to emotional resolution is measured in seconds; maximum skin conductance was measured in microSiemens; inter-beat interval was measured in milliseconds.*

### UNIVARIATE CORRELATIONS

We did not find any significant correlations between maximum SC amplitude and time to emotional resolution or subjective ratings of emotional intensity for positive, negative, or neutral pictures (Spearman’s rho < 0.13, *p* > 0.18) in either experimental condition. Similarly, no significant correlations were noted between IBI and time to emotional resolution or subjective ratings of emotional intensity for positive, negative, and neutral pictures (rho < 0.09, *p* > 0.39) in either experimental condition.

### TIME TO EMOTIONAL RESOLUTION

**FIGURE 2:**
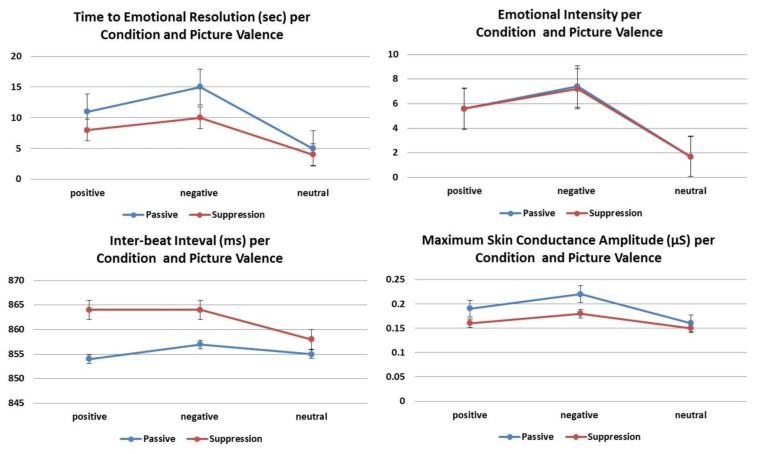
**The mean and SEs of subjective and physiological correlates of experiential suppression compared to passive viewing according to valence.** The effect of condition was significant (all *p* < 0.0001) for all variables except emotional intensity.

There was a significant main effect of condition [*F*(1,479) = 84.6; *p* < 0.0001]. Time to emotional resolution was significantly reduced in the suppression condition (*β* = –0.31, SE = 0.03, *p* < 0.0001; **Figure 2**). There was also a significant main effect of valence [*F*(2,479) = 296.7; *p* < 0.0001]. Regardless of condition, time to emotional resolution was longer for emotionally valenced compared to neutral pictures [negative: *β* = 0.98, SE = 0.04, *p* < 0.0001; positive: *β* = 0.68, SE = 0.04, *p* < 0.0001] and for negative compared to positive pictures (*β* = 0.31, SE = 0.04, *p* < 0.0001). None of the other measures showed a significant main effect and no significant interactions were found (all *p* > 0.05).

### SUBJECTIVE RATINGS OF INTENSITY

Valence had a significant effect [*F*(2,475) = 1453.7; *p* < 0.0001] regardless of condition; negative pictures elicited higher ratings than positive ones (*β* = 1.60, SE = 0.11, *p* < 0.0001) while neutral pictures had the lowest ratings (versus negative: *β* = 5.58, SE = 0.11, *p* < 0.0001; versus positive: *β* = 3.98, SE = 0.11, *p* < 0.0001; **Figure 2**). There were no significant main effects of condition [*F*(1,475) = 0.78; *p* = 0.38; **Figure 2**], sex [*F*(1,93) = 0.01; *p* = 0.91], or age (*β* = 0.008, SE = 0.01, *p* = 0.41).

We also found significant main effects of the TPQ-RD (*β* = 0.05, SE = 0.02, *p* = 0.04) and the FCP (*β* = 0.016, SE = 0.008, *p* = 0.05) on valence but not condition. Higher TPQ-RD and FCP scores indicated higher subjective intensity ratings for negative and positive pictures (all *p* < 0.01). None of the other measures showed a significant main effect and no significant interactions were found (all *p* > 0.05).

### MAXIMUM SC AMPLITUDE

Maximum SC amplitude was reduced in the suppression condition [*F*(1,460) = 21.8; *p* < 0.0001; **Figure 2**]; the effect was most pronounced in women in whom experiential suppression reduced the maximum SC amplitude below that of the passive condition (*p* < 0.0001; **Figure 2**).

We also found a significant main effect of valence [*F*(2,460) = 10.5; *p* < 0.0001]. Regardless of condition, maximum SC amplitudes were greater for negative (*β* = 0.22, SE = 0.049, *p* < 0.0001) and positive pictures (*β* = 0.15, SE = 0.049, *p* < 0.01) compared to neutral ones but there was no difference between negative and positives pictures (*β* = 0.063, SE = 0.049, *p* = 0.58). None of the other measures showed a significant main effect and no significant interactions were found (all *p* > 0.05).

### INTER-BEAT INTERVAL

The IBI was longer during the suppression than the passive condition (*β* = 8.23, SE = 2.75, *p* < 0.01; **Figure 2**). There was also a significant main effect of valence [*F*(2,424) = 3.1; *p* < 0.05]. Regardless of condition, the IBI was longer for negative than neutral pictures (*β* = 8.17, SE = 3.31, *p* < 0.05); there were no differences between negative and positive pictures (*β* = 4.27, SE = 3.31, *p* = 0.59) and between positive and neutral ones (*β* = 3.90, SE = 3.31, *p* = 0.71). None of the other measures showed a significant main effect and no significant interactions were found (all *p* > 0.05).

## DISCUSSION

We found that experiential suppression during the processing of emotive visual stimuli was associated with decreased duration of the emotional experience and decreased arousal as indexed by reduced maximum SC amplitude and longer IBIs. These findings were independent of age, valence, personality traits, hedonic capacity, and anxiety.

In our study, intensity ratings were obtained after stimulus offset and following self-reported resolution of the emotional experience. Therefore, they reflect participants’ evaluation of their recent emotional experience. Neuroimaging studies have highlighted the importance of the amygdala, the hippocampus, and the ventral prefrontal cortex (PFC) in emotional experience and regulation (Critchley et al., 2000; Williams et al., 2001; Patterson et al., 2002; Craig, 2003; Nagai et al., 2004; Steele and Lawrie, 2004; Garrett and Maddock, 2006). The evidence emerging suggests some degree of functional specialization within the ventromedial PFC-amygdala and ventrolateral PFC-hippocampal networks (Williams et al., 2001; Patterson et al., 2002). The ventromedial PFC-amygdala network processes primarily visceral information concerning arousal (Williams et al., 2001). This accords with earlier lesion studies in which electrodermal activity, measured using SC, was associated with the integrity of the ventromedial PFC (Tranel and Damasio, 1994). In contrast, the function of the ventrolateral PFC-hippocampal network has is primarily to evaluate or monitor emotion (Hariri et al., 2000; Lane, 2000). Garrett and Maddock (2006) showed that the amygdala were engaged only during the viewing of the emotive images while the ventrolateral PFC and the hippocampus were recruited both during perception and following stimulus offset. Subjective ratings of emotional intensity correlated with ventrolateral PFC activation suggesting that activity within this region is associated with evaluative appraisal of the elicited emotional experience (Garrett and Maddock, 2006). Moreover, evaluative appraisal of the elicited emotional experience appears independent of arousal as it can occur in the absence of SC responses (Williams et al., 2001). Consistent with these findings, there was no correlation between intensity ratings and either SC (maximum SC amplitude) or time to resolution in this study confirming that neither of these measures contributes to reflective evaluation.

As predicted by the negative bias hypothesis (Peeters and Czapinski, 1990), negative pictures elicited the most intense physiological and emotional responses. Specifically, we found that perseverance of emotional responses (time to emotional resolution) and arousal (SC maximum amplitude) were higher for negative compared to positive and neutral images. Perseverance of emotional responses is a key dimension of affective morbidity. All operationalized diagnostic systems include a duration criterion since perseverance of affective, mainly negative, responses defines abnormal emotional states [World Health Organization (WHO), 1992; American Psychiatric Association (APA), 2013]. Perseverance of negative responses is also a key risk factor for affective morbidity. In adults, the duration of negative responses following minor daily stressors is predictive of self-reported anxiety and depressive disorders (Charles et al., 2013). Similarly, Neumann et al. (2011) showed that 13–14-year-old adolescents with difficulties in regulating the duration of their negative emotional responses were significantly more likely to experience anxiety disorders and depressive symptoms. In this context, enhancing the ability to voluntarily suppress the duration of emotional experiences may have therapeutic and preventive potential.

Our results suggest that emotional suppression is not a unitary mechanism. Expressive suppression (i.e., the suppression of emotion-related behaviors) has been consistently associated with increased cardiovascular activation and arousal (Webb et al., 2012). In contrast, our findings suggest that experiential suppression may have the opposite effect. We found that IBIs were longer during experiential suppression than passive viewing. The IBI is dynamically regulated by the sympathetic and parasympathetic autonomic nervous systems; changes in IBI reflect a complex interaction between these two systems (Billman, 2011). In general, however, longer IBIs are associated with greater parasympathetic activity. At the same time, arousal was reduced by experiential suppression compared to passive viewing as indicated by lower maximum SC amplitude. Similar subjective and physiological changes have also been observed during relaxation and following successful psychological interventions resulting in improved emotion regulation (Linehan, 1993).

Personality traits had a limited influence on our results with the exception of measures relating to reward processing as reflected by reward dependent temperament derived from the TPQ and by hedonic capacity as measured by the FCP scale. Higher reward dependence and greater hedonic capacity were independently associated with elevated ratings of emotional intensity for negative and positive pictures. This finding indicates that the intensity of response to emotive stimuli is partly based on the perception of their hedonic value (Der-Avakian and Markou, 2012). Our results reinforce previous findings linking reward processing to increased intensity of emotional responses to positive and negative stimuli (Lundqvist, 2008). Reward processing, however, does not seem to influence the duration of emotional responses and hence it showed no interaction with experimental condition.

This study benefits from a large community-based sample of healthy adults, with equal representation of men and women, who were well characterized on multiple dimensions. IBI and SC activity were averaged over a sustained period of time (i.e., stimulus offset till the end of the subjective emotional experience). Although this type of analysis may obscure multiphasic responses that could be relevant to emotion regulation it is informative about sustained trends. We did not ask participants how they effected suppression and we are therefore unable to comment as to whether they employed particular cognitive strategies. Future studies will need to focus on the reproducibility of these findings across paradigms with different mood induction approaches and the relevance of experiential suppression to treatment interventions.

## AUTHOR CONTRIBUTIONS

Mathieu Lemaire conducted the data analysis and drafted early versions of the manuscript. Wissam El-Hage contributed to data interpretation and to drafting the manuscript. Sophia Frangou was responsible for the conception and design of the study, oversaw data acquisition and analysis, and contributed to drafting the manuscript. All authors read and approved the final manuscript.

## Conflict of Interest Statement

The authors declare that the research was conducted in the absence of any commercial or financial relationships that could be construed as a potential conflict of interest.

## Footnotes

1 http://www.psycho.uni-duesseldorf.de/abteilungen/aap/gpower3/

2 http://www.psylab.com

3 http://www.psychlab.com

4 http://www.R-project.org
